# Draft Genome Sequence of Multidrug-Resistant Pseudomonas protegens Strain 11HC2, Isolated from Marine Plastic Collected from the West Coast of Norway

**DOI:** 10.1128/MRA.01285-20

**Published:** 2021-01-14

**Authors:** Vera Radisic, Bjørn Tore Lunestad, Monica Sanden, Michael S. Bank, Nachiket P. Marathe

**Affiliations:** a Institute of Marine Research, Bergen, Norway; b Department of Biological Sciences, University of Bergen, Bergen, Norway; Georgia Institute of Technology

## Abstract

We report the draft genome sequence of multidrug-resistant Pseudomonas protegens strain 11HC2, isolated from polypropylene collected from the water column near a beach in Øygarden, Norway. The genome sequence is 6,861,219 bp long, with a G+C content of 63.4%. Strain 11HC2 is resistant to cefotaxime, ampicillin, trimethoprim, and chloramphenicol.

## Abstract (precis)

We report the draft genome sequence of the multidrug-resistant Pseudomonas protegens strain 11HC2, isolated from polypropylene collected from a water column near a beach in Øygarden, Norway. The genome sequence is 6,861,219 bp in length, with a G+C content of 63.4%. Strain 11HC2 is resistant to cefotaxime, ampicillin, trimethoprim, and chloramphenicol.

## ANNOUNCEMENT

*Pseudomonas* spp. are diverse and widespread in the environment (1). Many of these are known to cause infections in humans, animals, and plants (2, 3), whereas others are used for bioremediation (4). In this study, we report the whole-genome sequence of multidrug-resistant Pseudomonas protegens strain 11HC2, isolated from plastic debris collected from the intertidal zone (submerged in water) near a beach (60°29*′*57.6*″*N, 4°55′05.3*″*E) in Øygarden, Norway (5).

The method used for isolation and identification of the strain is described by Radisic et al. (5). Briefly, strain 11HC2 was isolated by spreading serial dilutions of suspensions prepared from plastic-associated biofilms onto Mueller-Hinton agar plates containing cefotaxime (10 μg/ml) and incubating it aerobically at 25°C for 24 to 48 h. This strain was purified by restreaking it onto Mueller-Hinton agar with ampicillin (100 μg/ml) and incubating it at 30°C for 24 h. Identification was performed using the Bruker MALDI Biotyper at the Institute of Marine Research (Bergen, Norway) using the MALDI Biotyper database. A culture grown overnight at 30°C was used for determining the MICs for cefotaxime, tetracycline, ciprofloxacin, ampicillin, meropenem, streptomycin, trimethoprim, gentamicin, imipenem, and chloramphenicol using an Etest following the manufacturer’s instructions (bioMérieux, Paris, France). Strain 11HC2 had a MIC of >32 μg/ml for cefotaxime and trimethoprim and >256 μg/ml for ampicillin and chloramphenicol. Genomic DNA was extracted from a fresh culture (grown aerobically at 30°C overnight) using a QIAamp fast DNA stool minikit (Qiagen, Hilden, Germany), following the manufacturer’s instructions. A NanoDrop 1000 spectrophotometer and Qubit double-stranded DNA (dsDNA) broad-range (BR) assay kit (Thermo Fisher Scientific, Waltham, MA, USA) were used for quantification of the extracted DNA. Genomic DNA (at 4°C) was sent for sequencing to the Norwegian Sequencing Centre (Oslo University Hospital, Ullevål, Oslo, Norway). A 2S Turbo DNA library kit (Swift Biosciences, Ann Arbor, MI, USA) was used for preparing sequencing libraries. Sequencing was carried out on the MiSeq platform (Illumina, San Diego, CA, USA), using 2 × 300-bp chemistry. A total of 1,055,968 reads were obtained. Adapters were removed from the reads followed by quality trimming in BBDuk version 38.75 (https://jgi.doe.gov/data-and-tools/bbtools/bb-tools-user-guide/). After quality filtering, 946,456 reads were used for genome assembly (approximately 41-fold coverage). Sequences were assembled in SPAdes version 3.13.0 (6). Default parameters were used for all software unless otherwise mentioned. The genome assembly produced 88 contigs (length, >500 bp) with a total length of 6,861,219 bp, an *N*_50_ value of 189,533 bp, and a G+C content of 63.4%. The longest contig was 659,287 bp. The average nucleotide identity based on BLAST, calculated using JSpeciesWS (7), confirmed that strain 11HC2 belongs to Pseudomonas protegens. Genome annotation was performed using the National Center for Biotechnology Information (NCBI) Prokaryotic Genome Automatic Annotation Pipeline (PGAAP) (8), where 6,180 coding DNA sequences (CDS), 62 tRNAs, and 7 rRNAs were identified. Antibiotic resistance genes were screened using the CARD database version 3.0.7 (9) and the ResFinder database version 3.2 (10). Strain 11HC2 carries a class C β-lactamase, type B chloramphenicol O-acetyltransferase (*catB*), three different copies of dihydrofolate reductase, and a bifunctional aminoglycoside phosphotransferase/ATP-binding protein.

### Data availability.

This whole-genome shotgun project has been deposited in DDBJ/ENA/GenBank under the accession number JADFCN000000000. The raw sequencing data have been deposited under the SRA accession number SRR12929845 and the BioProject accession number PRJNA670160.
